# Device for Identifying the UV Emission Spectrum

**DOI:** 10.3390/s22134852

**Published:** 2022-06-27

**Authors:** Robert Jenő Kovács, Jenő-Zoltán Kovács, Lorant Andras Szolga

**Affiliations:** Optoelectronics Group, Basis of Electronics Department, Faculty of Electronics, Telecommunications and Information Technology, UTCN, 400114 Cluj-Napoca, Romania; robert-jeno.kovacs@outlook.com (R.J.K.); jeno-zoltan.kovacs@profitool.ro (J.-Z.K.)

**Keywords:** UV radiation, UV sensors, UV LED, Arduino, Bluetooth

## Abstract

Nowadays, the disinfection of classrooms, shopping malls, and offices has become an important part of our lives. One of the most effective disinfection methods is ultraviolet (UV) radiation. To ensure the disinfection device has the required wavelength spectrum, we need to measure it with dedicated equipment. Thus, in this work, we present the development of a UV spectrum detector capable of identifying UV wavelength spectrums, with a wide range of probes and the ability to transmit data to a PC for later evaluation of the results. The device was developed with four UV sensors: one for UV-A, one for UV-B, one for UV-C, and one with a wide range of detection of UVA, with a built-in transimpedance amplifier. An Arduino Nano development board processes all the acquired data. We developed a custom light source containing seven UV LEDs with different central wavelengths to calibrate the device. For easy visualization of the results, custom PC software was developed in the Processing programming medium. For the two pieces of electronics—the UV detector and calibration device—3D-printed housings were created to be ergonomic for the end-user. From the price point of view, this device is affordable compared to what we can find on the market.

## 1. Introduction

Disinfection has always been an essential field for research due to the varied needs for disinfection in hospitals, clinical, sanitary, and food production areas. With the COVID-19 pandemic, these places of use have been expanded to nearly all areas of everyday life.

Disinfection based on electromagnetic radiation from the ultraviolet (UV) range C, which is between 100 nm and 280 nm, requires electromagnetic radiation with wavelengths from 222 nm (1350 THz) to 265 nm (1131 THz). The corresponding energy is in the order of 5 × 10^−19^–2 × 10^−17^ J, which interacts with living tissue in the human body in a specific way.

The electromagnetic radiation from the UV-C spectrum offers efficient disinfection properties at the level of bacteria, germs, and viruses in sterilization processes (water, air, volume, surface, materials, components, food, etc.) Since the middle of the nineteenth century, it has been used with solar UV exposure, and starting in the middle of the twentieth century, it has been used widely with artificial UV generators [1,2,3,4,5,6,7,8,9,10].

From the electromagnetic radiation of the UV spectrum generated by the Sun, only partially filtered UV-A, almost completely filtered UV-B, and filtered UV-C reaches the Earth’s surface due to the ozone layer in the atmosphere. The possibility of exposure of living tissue to UV-C radiation, which is dangerous, comes from artificial radiation sources: e.g., lamps or UV LED, lasers, and electric arc welding.

The germicidal range of electromagnetic radiation in the UV-C spectrum has the peak for traditional germicidal activity at 265 nm. According to the latest research, this peak is at 222 nm. These UV-C radiations are absorbed by the DNA and RNA of microorganisms and destroy the structure of DNA and RNA, causing an inability to reproduce. A cell that cannot reproduce is considered dead since it cannot multiply the number of infected cells in a host. The process is also called antibacterial, germicidal, or anti-viral ultraviolet irradiation, proving its high efficiency against a broad spectrum of microorganisms (bacteria, germs, viruses).

Advantages of UV-C radiation disinfection are a short disinfection time, simplicity, no air flow, no room insulation, no chemicals, low maintenance, and a long service life. There are also limitations: it requires direct radiation, is inefficient in shaded areas, does not penetrate deep into living tissues or organic materials, and the irradiation distance decreases the yield. Thus, it is necessary to prevent and spread infections in public and private places by disinfecting rooms, surfaces, objects, and medical instruments, and improve the existing classical methods for disinfection and cleaning [11].

Directly measuring the UV radiation wavelengths of disinfection systems contributes deeply to the process by ensuring the user is in the efficient spectrum for disinfection.

Different approaches have been used to precisely measure the UV wavelength energies of a light source. All of them involve using some physical sensors developed especially for these applications. Registered patents use gas-type sensors [12] or semiconductor-type sensors to detect UV energy [13,14,15,16,17,18].

Regarding the semiconductor type of sensor, many UV detection devices have been developed. To obtain a high energy measurement accuracy, or at least the detection of each wavelength from the UV spectrum, the best solution is using a wide window optical detector combined with high-resolution selective filters or diffraction gratings. This concept is known as a spectrometer and involves high costs.

Affordable spectrometers can be implemented in the visible spectrum, as in [19], where a light-dependent resistor (LDR) was used to detect the wavelengths with a low resolution.

A spectrometer that detects only spectrum presence in ultraviolet-visible (UV-Vis) is detailed in [20]. The presence of 280 nm and 595 nm central wavelength spectrum emissions of the two LEDs was detected using a wide spectrum (200–1100 nm) OSD5.8−7Q photodetector in an interaction with biological protein probes.

A similar spectrometer to [20] was implemented in [21]. The 280 nm spectrum presence was detected using a PDU-G106B photodetector (200–320 nm) and the interaction of the UV emission of a T9H28C LED, at a peak wavelength of 280 nm, with a goat anti-human immunoglobulin (GaHIgG) probe.

In work [22], a UV energy measurement device is presented for emissions from the Sun. The device was developed using an ML8511 photodetector module, incorporating a broad spectrum photodiode and a transimpedance amplifier. It was calibrated for use in different regions of the globe.

In all the works above, the small resolution spectrometers can be considered spectrum detectors from the operational point of view. They use narrow-spectrum emission LEDs as reference light sources and different agents (proteins or solutions) as indirect filters.

The interest in the disinfection process and the development of new UV LEDs in this direction is an active research field, as shown in recent works [23,24,25,26,27,28,29,30,31].

In our work, we extended the spectrum detection in the field of UV emissions to three main regions—UV-A, UV-B, and UV-C—using four different spectrum detection photodiodes and seven UV LEDs with narrow-spectrum emission for the calibration process of the device.

## 2. System Description

### 2.1. System Overview

We present a UV wavelength-measuring device called the KS UV METER, designed to detect the presence of the three UV radiation spectrums found in the 200–400 nm range. It directly measures the wavelength of ultraviolet radiation in the 400–315 nm range for UV-A radiation, 315–280 nm for UV-B range radiation, and 280–200 nm for UV-C range radiation.

The development and implementation of the final device involve two hardware parts (the measuring device and the calibrator) and two software parts (the device firmware and custom PC software for the interpretation of the data and results.

**Figure 1:**
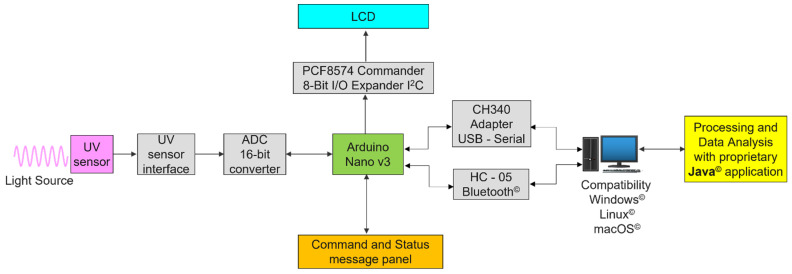
Block diagram of the device.

The measuring process flow is presented schematically in Figure 1.

### 2.2. Hardware

#### 2.2.1. UV Spectrum Detection Device

**Figure 2:**
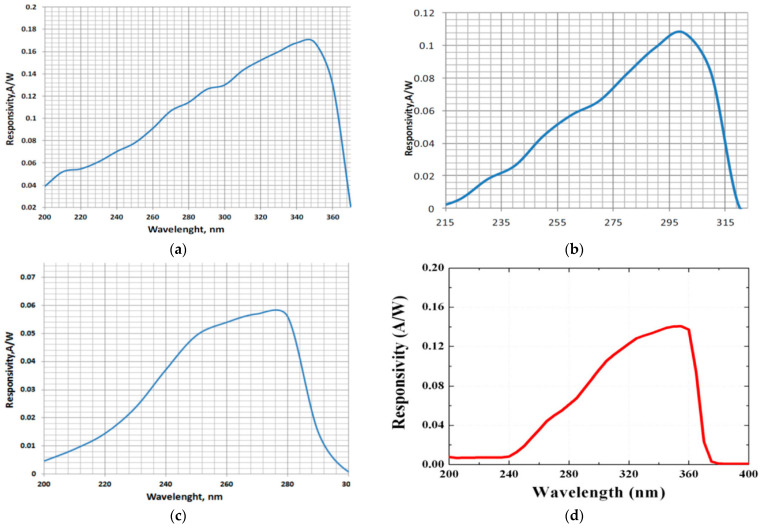
Spectral response of the photodetectors: (**a**) D1 (SD008-2151-112), (**b**) D2 (SD008-2161-112), (**c**) D3 (SD008-2171-112), (**d**) IC1 (GUVA-S12SD). (All the graphs were extracted from the manufacturer’s datasheet).

When a flux of UV radiation drops on the UV sensor (photodiode or modular sensor), it ionizes the semiconductor, and the electrons start to flow. Energy is converted from UV radiation to electrical power. The generated photocurrent is proportional to the incident optical power and depends on its wavelength. Thus, to accurately detect the energy present in one of the three UV spectrums, we chose three UV sensors (SD008-2151-112, SD008-2161-112, and SD008-2171-112) with high sensitivity in each of these spectrums and, at the same time, to act as a cutting filter for the other spectrums. To detect the presence of the UV spectrum from 240 nm to 370 nm, we used the modular GUVA-S12SD photodetector with a built-in transimpedance amplifier. The spectral response of these photodetectors is presented in Table 1 and Figure 2.

**Figure 3:**
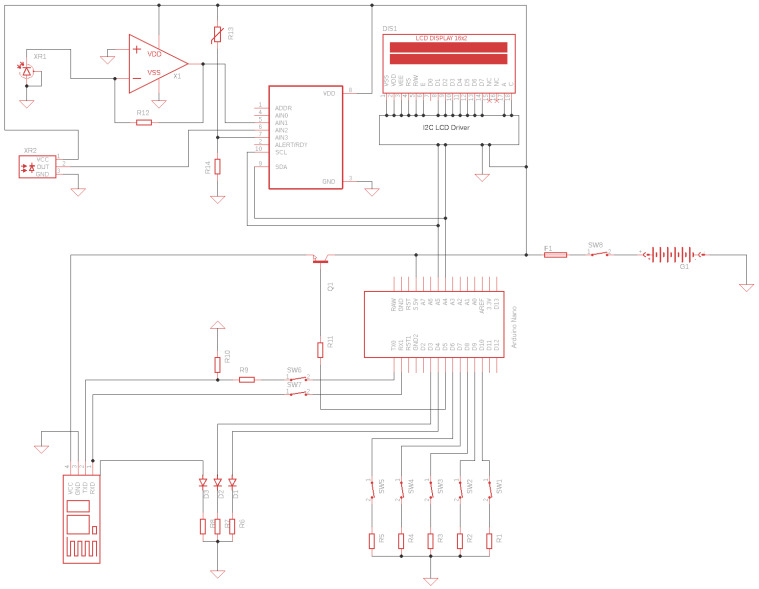
Electronic circuit for the UV measurement device.

All four UV sensors are Schottky-type photodiodes with high responsivity, low dark current, and good visibility blindness. We used reverse polarization in the circuit for the D1, D2, and D3 photodetectors by connecting the anode to the ground and the cathode at the non-inverting input of the precision instrumentation amplifier, type OA1ZHA (Figure 3). The amplifier is used in transimpedance configuration and provides low input and output resistance to short-circuit the UV sensor signal source. This signal source, with proper resistance, behaves as a constant current source, dependent only on the wavelength and the intensity of the measured incident UV radiation and achieving a linear ratio between the current generated by the UV sensor and the output voltage of the operational amplifier powered by the 3.3 V internal voltage stabilizer of the Arduino Nano development board.

The ambient temperature sensor was implemented with a negative temperature coefficient (NTC) thermistor with a negative temperature characteristic of 1 kΩ.

The signal from the output of the transimpedance amplifier is digitized by a 16-bit ADS1115 precision digital-analog converter. The ADS1115 has four analog inputs, adjustable amplification, internal reference voltage, an internal clock, a programmable internal comparator that can transmit a signal on a dedicated output, and a programmable interface inter-integrated circuit protocol (I^2^C) that allows several integrated circuits of the “slave” type to communicate with one or more integrated circuits of the “master” type on the same protocol. We chose to use this option because the Arduino Nano board can use the I^2^C communication protocol for both the 2 × 16 LCD display and the precision digital–analog converter through pins A4 and A5—the only analog pins of the development that can be used for digital communication (SDA—the data transmission line and SCL—the internal clock synchronization line).

The ADS1115 collects analog data from the output of the transimpedance amplifier at its analog input A3, from the output of the IC1 modular sensor at its analog input A2, and from the thermistor at its analog input A1. It can generate a generous 65.536 sampling levels, compared to the only 1024 levels provided by the 10 bit ADC from the Atmega328 incorporated into the Arduino Nano board.

**Figure 4:**
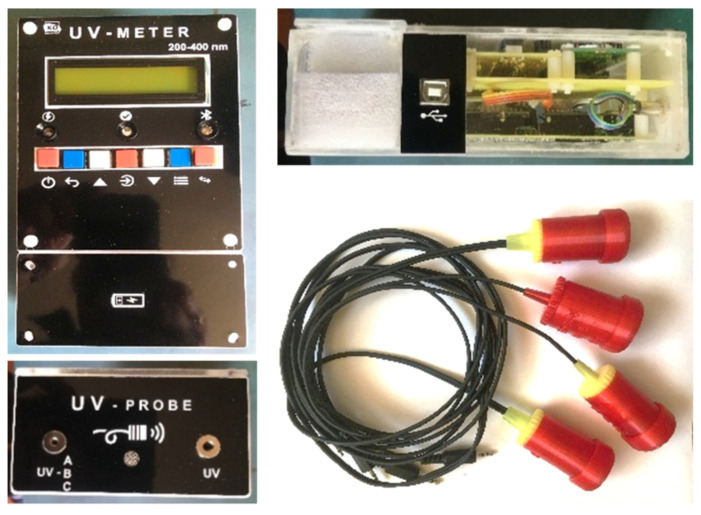
UV spectrum detector device in housing with probes.

The spectrum detection device (Figure 4) was mounted on a transparent acrylic glass (perspex) housing with 3 mm walls for visualizing the interior components and laser-engraved labels for the inscriptions on the front and side connection panels. The design was done in Corel Draw.

**Figure 5:**
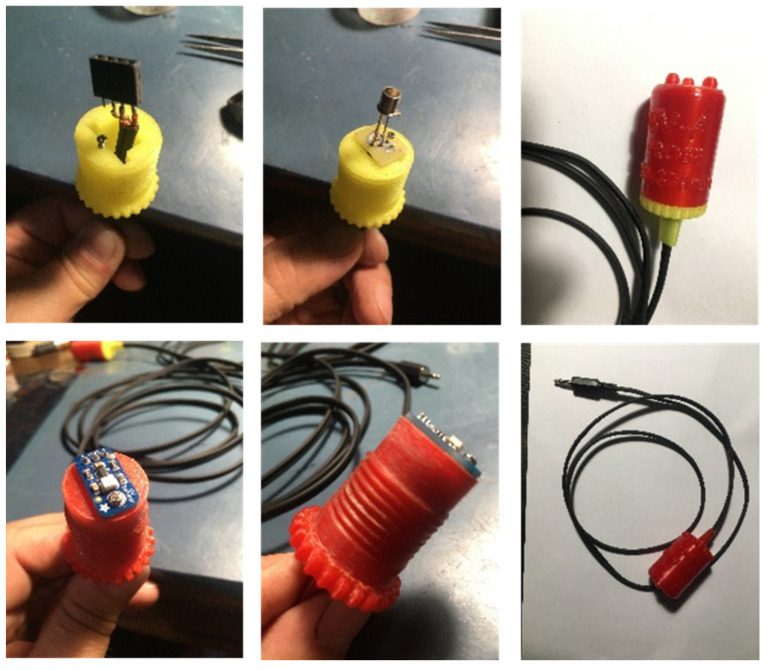
UV sensor probes mounted on 3D-printed enclosures and wired up for Arduino connection.

Precision UV probe housing D1, D2, D3, and IC1 and calibrator housing were designed in Solid Works and printed with a Flashforge Createbot F43 3D printer (Figure 5).

The wiring diagram of the wavelength-measuring device and the UV calibrator circuit was designed in Eagle. The corresponding layout of these electronic circuits was done in the Sprint Layout program.

#### 2.2.2. The Calibrator Device

**Figure 6:**
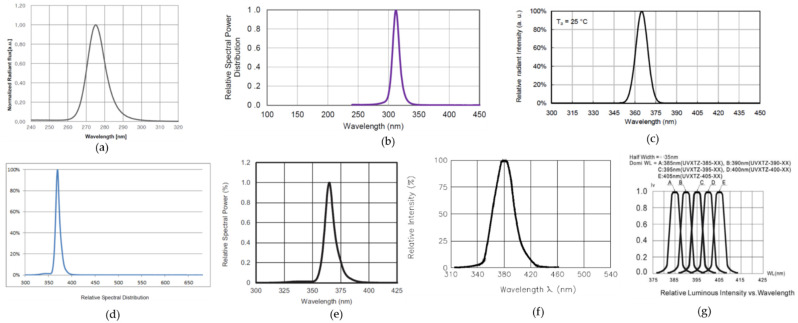
Optical spectrum emission of the seven UV LEDs from the calibrator device: (**a**) #1 (CUD7QF1A); (**b**) #2 (PB2D-UCLA-KB); (**c**) #3 (ATS2012UV365); (**d**) #4 (LTPL-C034UVH365); (**e**) #5 (VLMU1610-365-135); (**f**) #6 (VAOL-5GUV8T4); (**g**) #7 (UV5TZ-395-30). (All the graphs were obtained from the manufacturer’s datasheet).

The calibration process of the measuring device is done through seven standard UV LEDs that radiate wavelengths between 275 nm and 395 nm. Thus, the device with the seven LEDs is called the calibrator. The detailed electro-optical parameters of these LEDs are given in Table 2 and Figure 6.

The LEDs were chosen for a narrow half-width spectrum and optical power generated by a few milliwatts. From Table 2, it can be observed that all the LEDs have a half-width spectrum close to 10 nm. The area of the light-emitting semiconductor was not provided in the manufacturer’s datasheet, and we considered the general case of such devices to have an area of emission under 1 mm^2^ (hundreds of µm^2^).

**Figure 7:**
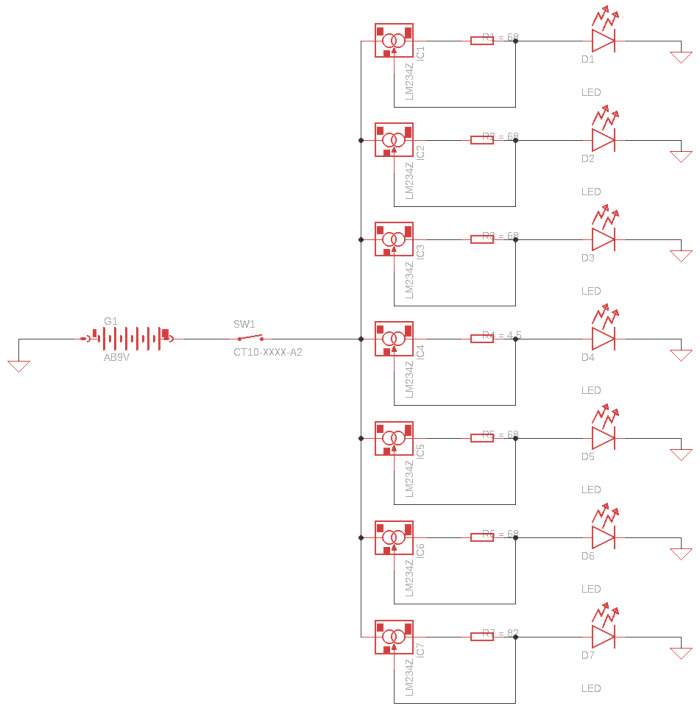
Electronic circuit of calibrator.

**Figure 8:**
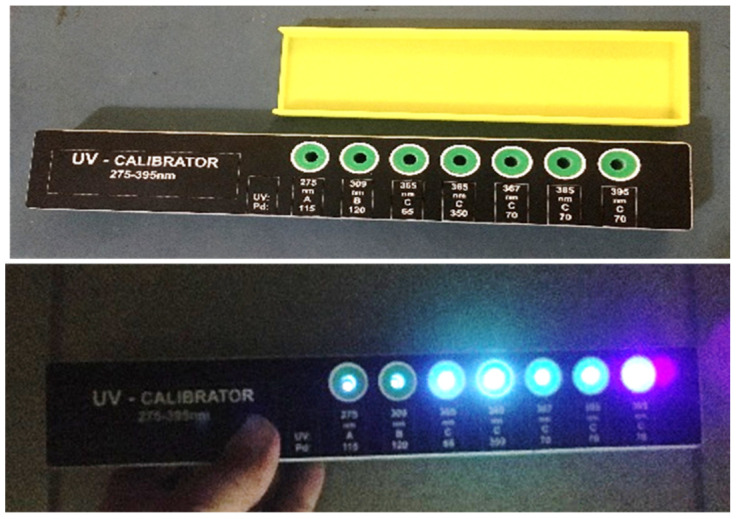
Assembled calibrator with UV LEDs on.

The LEDs are supplied using a constant current generator with an LM317 voltage regulator (Figure 7). The input of the voltage regulator is connected to the positive terminal of the power supply via the “start” button of the calibrator. The output of the voltage regulator is connected to the anode of the UV LED through resistance for current regulation. The adjustment terminal (ADJ) for the continuous monitoring of the voltage drop of 1.25 V on the current control resistor ensures the continuous regulation of the current passing through the UV LED. With such a connection, the LM317 acts as a voltage regulator and a constant current generator for the UV LEDs. To fulfill the correct operation of the LM317, the voltage difference between the input and output voltage must be at least 3 V (Equation (1)). The output voltage includes the 1.25 V (V_ADJ_) regulated voltage and the voltage drop (V_F_) across the UV LED, which is in the range of 3.5 V–4 V (Equation (2)). Based on the above consideration and the extreme case for the UV LED forward voltage, a minimum input voltage of 8.25 V is necessary (Equation (3)). Thus, we considered using a 9 V battery as the power supply for the calibrator. An alkaline battery can work for around three hours, and with a zinc–carbon battery, for about two hours continuously (Figure 8). Table 3 presents the values for the resistances and the constant current generated with the LM317 for each UV LED.

(1) $$V_{in}-V_{out}=3V$$

(2) $$V_{out}=V_{ADJ}+V_{F}\;$$

(3) $$V_{in\_min}=3V+1.25V+4V\;$$

### 2.3. Software

#### 2.3.1. Firmware

An Arduino Nano open-source development board controls the spectrum detector device. The measured data, identified spectrum, temperature, and UV sensors are displayed on a 2 × 16 matrix LCD.

The Arduino board communicates through the serial communication port (Rx/Tx) with its converter, type CH340 (which performs the serial conversion—USB), transmitting the data to the PC. Alternatively, using the Bluetooth serial port, type HC-05, the data can be transmitted through wireless communication at a short distance (0.5–100 m max.). The control and adjustment buttons (MC) and three LEDs as signaling elements (ML) are mounted on the device’s front panel.

Powering the device with a 9 V battery, preferably alkaline or zinc–carbon, with a fuse of 200 mA for short-circuit protection, ensures the continuous operation of approximately four (zinc–carbon battery) to six hours (alkaline battery).

A “FLASH” memory of 32 kb from the ATmega328 microcontroller contains the program that automatically loads the boot operating system (bootloader), which occupies 2 kb together with the dedicated program for operation and is accessed only at startup by reading, operating at an internal clock frequency of 16 MHz. An 8 kb static random-access memory (SRAM) device is loaded after starting the circuit and is the program for performing calculations and decisions after measurements, operating at 8 MHz, which is half the frequency of the internal clock. For data storage, 1 kb of electrically erasable programmable read-only memory (EEPROM) is used. This is necessary for calculations after measurements and preset action (e.g., the calibration of sensor parameters, commands for various preset settings, lighting or without lighting 2 × 16 LCD, etc.), with a write speed of approx. 4 ms (250 Hz), and for readings of approx. 16 MHz/4 = 4 MHz.

**Figure 9:**
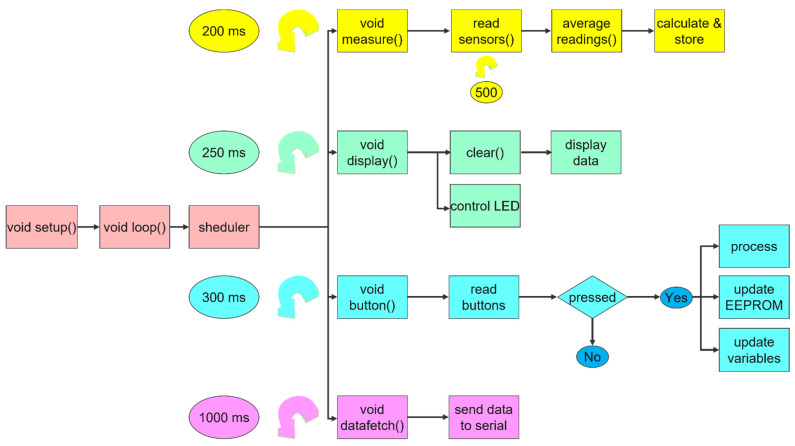
Logic diagram of the firmware.

The logic diagram of the operational built-in functions of the firmware program is presented in Figure 9.

The Arduino Nano can execute only a single threading process during its operation (e.g., when reading a sensor, it cannot send data to the 2 × 16 LCD simultaneously). Thus, the program has been split into several executable functions that are accessed cyclically and continuously in the main function, called *void loop (vl)*:

- The first function, void measure (vm), is called at equal time intervals of 200 ms and ensures the performance of the UV measurements and ambient temperature by reading the measurement data from the UV and ambient temperature sensors. The measured data are compared with the data from the EEPROM memory to decide the subdomain (UV-A, UV-B, UV-C) of radiation of the UV spectrum.
- The second function, called *void button (vm)*, is called at equal time intervals of 300 ms and follows the commands of the control buttons, based on which it navigates in the program menu (MENU). Each menu screen has a code, based on which the set task is executed.
- The third function, called *void display (vm)*, is called at equal intervals of 200 ms and rewrites (REWRITE) the LCD 2 × 16, deleting and rewriting all the information for display, as well as ensuring the coupling or decoupling of the green LED to indicate the possibility of taking measurements (OK).
- The fourth function, called *void data fetch (vd)*, is called at equal intervals of 2 s and sends the measurement data through the serial communication port (Rx/Tx) by the comma separation method (comma-separated values), with a semicolon at the end of a series of data (e.g., 278, 3, 25, 31 indicates that the UV wavelength was measured at 278 nm in the UV-A subdomain (no. 3 means subdomain A, according to the coding), the measured ambient temperature value was 25 °C, the sensor type code used was 31 (no. 31 means the UV-A sensor type was SD008-2151-112), and the semicolon (;) at the end means the end of the measurement).

The void setup (vs) function runs outside of the main void function and only once at the start of the circuit for primary function initialization (e.g., writes the initial message on the 2 × 16 LCD: UV meter ver. 1.4). It reads from the EEPROM memory the stored data necessary for the calculations performed in the following measurements for evaluation and preset action (e.g., sensor characteristic information, whether to turn on the 2 × 16 LCD lighting, etc.).

#### 2.3.2. PC Program

**Figure 10:**
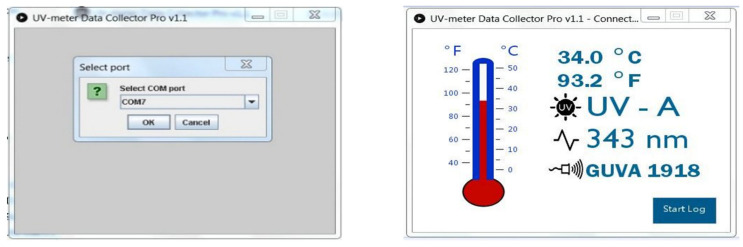
UV-meter Data Collector Pro v1.1 PC data logging software.

The program is called UV-meter Data Collector *PRO v1.1.* It was developed in the open-source Processing language and can be installed in the Windows, Linux, and Mac OS operating systems. It was created to store and evaluate the measured data of a UV meter. After launching the program and choosing the appropriate COM port for the UV device connection, it displays the main menu and waits for the data packets of measurements to be received via the Rx/Tx serial port (via wired connection by USB cable or via Bluetooth connection). The data are displayed in numerical values and graphically (bar graph). The errors of the data packet of transmission are not displayed on the screen, appearing only as an information error (ERROR), or the existence of the connection with the computer is displayed, and/or when not receiving data for 5 s (Connected/Disconnected) (Figure 10).

*PRO v.1.1.* creates a file with the extension “.csv”. This file contains the measurement data (wavelengths, ambient temperature, date, time, UV spectrum A-B-C, sensor type). It can be opened directly in the data management program Excel within Microsoft Office and processed as such (statistical, graphical).

## 3. Measurements, Results, and Discussion

To have a reference point for the sensitivity of all the UV sensors using the radiant power emitted by the UV LEDs, we used a monochromator as a controllable light source that can cover a part of the UV spectrum.

**Figure 11:**
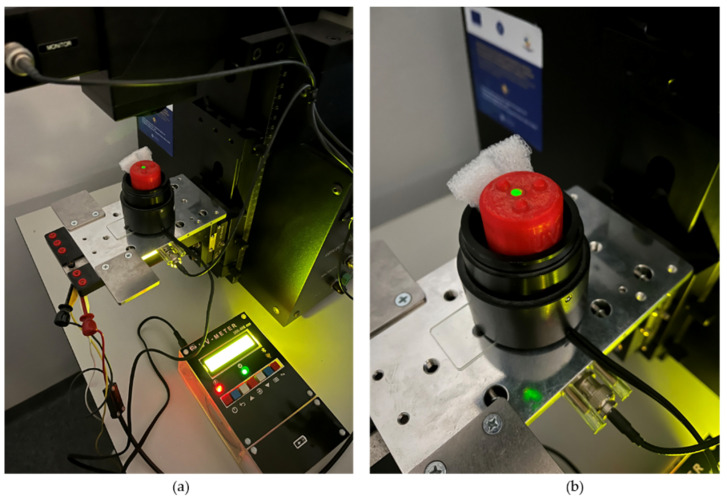
Testing the IC1 sensor with the monochromator (the emission wavelength is 555 nm): (**a**) view with the measuring device in action; (**b**) close-up view on the sensor probe.

We used a Newport IQE-200B monochromator to test the sensitivity of the IC1 sensor (Figure 11). We used this sensor because it presents sensitivity for the emission spectrum of 350 nm to 400 nm, which can be covered by the monochromator’s light source of 350–1100 nm. The wavelength accuracy of the monochromator is ±0.5 nm. The 100 W Xenon lamp (model #6257) incorporated in the monochromator generates a constant irradiance of 100 mW/m^2^ for each wavelength to a maximum distance of 0.5 m. The smallest concentrated spot size generated by the monochromator is 0.8 mm× 1 mm (0.8 mm^2^) at 74 mm [32]. The active area of the photodiode sensors given in the manufacturer’s datasheet is 0.076 mm^2^. In these conditions, the monochromator will generate irradiance values of 100 mW/0.8 mm^2^ and 9.5 mW/0.076 mm^2^.

The measured results depend directly on the intensity of the UV radiation flow, so the angle at which the incident UV radiation falls on the UV sensors is essential.

The maximum values of the measurements are reached in a perpendicular arrangement of a UV sensor directed towards a flux of UV radiation.

**Figure 12:**
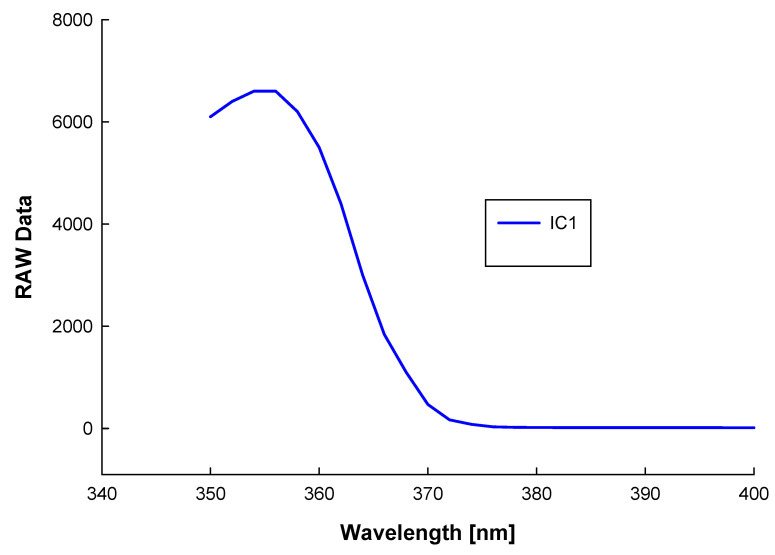
Measurements with the IC1 sensor under monochromator light source exposure.

The UV measurements taken with the monochromator as a light source are presented in Figure 12, where the “RAW data” represents the number of sampling levels read by the ADS1115.

**Figure 13:**
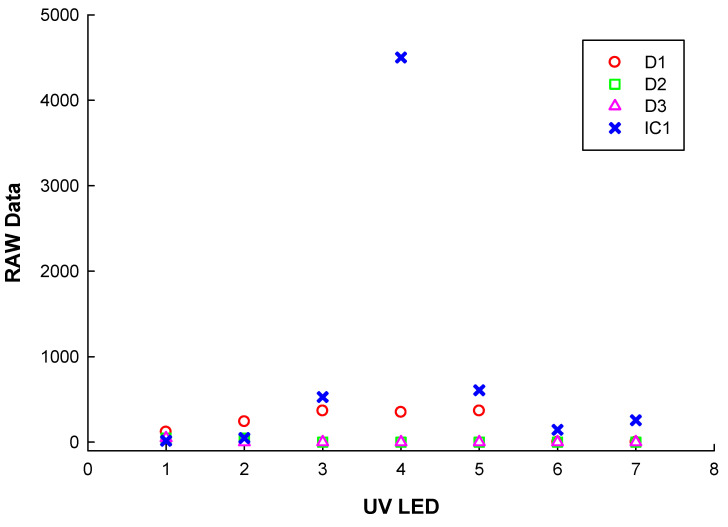
Measurements with the sensors under the exposure of the UV LEDs from the calibrator circuit.

For the UV measurements with the UV LED light sources, we considered that their large radiation angle at a considerable distance will significantly reduce their irradiance. Thus, we placed them in front of the sensors at a distance small enough (a few millimeters). At this distance, the irradiance can be considered proportional to the surface of the radiance of the LED. The sensors’ housing has a dotted spacer to keep the same distance from the UV LEDs to the sensors. With these considerations and the data presented in Table 2 and Table 3, the optical power emitted by the LEDs reaching the sensors should be as presented in Table 4. The “RAW data” measurements taken with the sensors using the UV LEDs are presented in Figure 13.

Analyzing the measurements results obtained with the monochromator and the UV LEDs along with the data from Table 1, Table 2 and Table 4, we obtained the following:

- At 355 nm and an irradiance of around 9.5 mW/0.076 mm^2^ on sensor IC1, we measured 6600 raw data;
- By exposing sensor IC1 to UV LED 4 with a central wavelength of emission of 365 nm and a half-width spectrum of 10 nm, we measured 4500 raw data;
- With the above two measurements (monochromator and UV LED 4) on IC1, we could estimate a real irradiance of around 6.5 mW, which reaches this sensor. This means that only 20% of a radiant flux of 30 mW/0.076 mm^2^ reaches the sensors’ surface. Thus, even using a very small distance between the UV LEDs and the sensors, significant radiant power is lost because of the large angle of emission of the UV LEDs;
- The plotted measurements in Figure 13 show the excellent filtering of the sensors in the undesired spectrums. Thus, they will react only to the spectrum where they are very sensitive, as presented in Table 1 and Figure 2;
- The measurements obtained with the low-power emission LEDs (1, 2, 3, 5, 6, and 7) prove that the sensors do not react to such low UV emissions.

**Figure 14:**
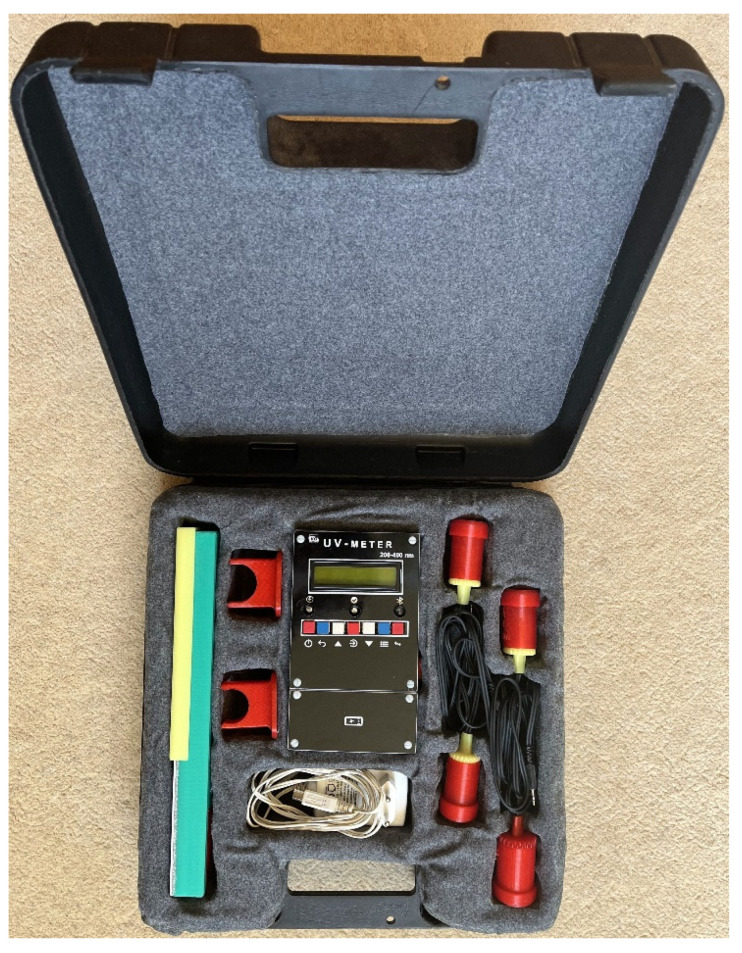
The whole setup.

Figure 14 presents the measuring device, probes, and calibrator in a suitcase.

## 4. Conclusions

The measured results depend directly on the intensity of the UV radiation flow, so the angle at which the incident UV radiation falls on the UV sensor of the device is essential. The maximum values of the measurements are reached with the perpendicular arrangement of a UV sensor directed towards a flux of UV radiation.

Similar devices are available on the market, but with costs above 1000 EUR, whereas our prototype can be built for under 200 EUR. Performance characteristics of different products cannot be directly compared because the available measurement devices measure UV radiation intensity and power in fixed or limited wavelength regions. In contrast, our device can only detect the presence of one of the subdomains of the UV spectrum.

The overall technical specifications of the system are as follows:

- Universal input: 240–380 nm of one domain of measurement;
- High precision input: UV-A/B/C, 200–400 nm, with three subdomains of measurement. Subdomains of measurement include UV-A: 315–400 nm; UV-B: 280–315 nm; UV-C: 200–280 nm;
- Measure interval: 200 ms;
- Adequate time of measurement: 1.16 ms;
- Display of arithmetical average of 50 measurements;
- LCD 2 × 16 dotted-shape matrix display with 2 × 16 characters (numbers or letters), with a refresh rate of 200 ms;
- Measurement send time to PC: 2 s;
- Measured data transmission: USB cable/Bluetooth;
- Standalone measuring device with a 9 V battery power supply (type 6F22);
- Dimensions: 103 mm × 60 mm × 173 mm;
- Weight: 392 g (without battery), 428 g (with battery);
- High-precision probes D1 (UV-A), D2 (UV-B), D3 (UV-C), diameter: 27 mm, length: 64 mm, cable: 950 mm, weight: 40 g (with cables and connectors);
- Probe UV of large band UV (IC1), diameter: 31 mm, length: 63 mm, cable: 950 mm, weight: 49 g (with cables and connectors);
- Calibrator: 45 mm × 30 mm × 279 mm, weight: 277 g (without battery), 313 g (with battery);
- Operating temperature: +5–+40 °C/0–45 °C;
- Operating humidity: 80% to 20 °C;
- Operating atmospheric pressure: 800–1060 mbar.

## Figures and Tables

**Table 1:** Spectral parameters of the UV sensors.

Photodetector	Purpose	Reference	UV Spectrum (nm)	Peak Wavelength for Responsivity (nm)	Responsivity (A/W)
SD008-2151-112	UV-A	D1	220–370	350	0.18
SD008-2161-112	UV-B	D2	240–320	300	0.1
SD008-2171-112	UV-C	D3	220–280	270	0.06
GUVA-S12SD	General UV	IC1	240–370	360	0.14

**Table 2:** Electro-optical parameters of the UV LEDs used in the calibrator device.

#	UV LED	Peak Wavelength, λp (nm)	Radiant Flux (mW)	IF Current (mA)	Spectrum Half-Width, Δλ (nm)	Radiant Angle 2 θ1/2 (deg)
1	CUD7QF1A	275	2	20	11	125
2	PB2D-UCLA-KB	309	3.3	20	12	120
3	ATS2012UV365	365	13	20	10	150
4	LTPL-C034UVH365	365	720	500	10	130
5	VLMU1610-365-135	367	23	20	8	130
6	VAOL-5GUV8T4	385	*	20	40	30
7	UV5TZ-395-30	395	40	15	35	30

* Values given in mcd.

**Table 3:** Constant current conditions for each UV LED in the calibrator circuit.

LED #	1	2	3	4	5	6	7
Resistance (Ω)	68	68	68	4.5	68	68	68
Current (mA)	18	18	18	277	18	18	18

**Table 4:** Evaluated irradiance on the sensors using the UV LEDs from the calibrator circuit.

LED #	1	2	3	4	5	6	7
Irradiance (mW)	0.15	0.25	0.99	30	1.75	0	3
